# Necrotizing fasciitis in patients with diabetes mellitus: clinical characteristics and risk factors for mortality

**DOI:** 10.1186/s12879-015-1144-0

**Published:** 2015-10-13

**Authors:** Nai-Chen Cheng, Hao-Chih Tai, Shan-Chwen Chang, Chin-Hao Chang, Hong-Shiee Lai

**Affiliations:** Department of Surgery, National Taiwan University Hospital and College of Medicine, 7 Chung-Shan South Road, Taipei, 100 Taiwan; Department of Internal Medicine, National Taiwan University Hospital and College of Medicine, 7 Chung-Shan South Road, Taipei, 100 Taiwan; Department of Medical Research, National Taiwan University Hospital and College of Medicine, 7 Chung-Shan South Road, Taipei, 100 Taiwan

**Keywords:** Necrotizing fasciitis, Diabetes mellitus, Mortality, Risk factor, Soft-tissue infection

## Abstract

**Background:**

Necrotizing fasciitis (NF) is a rapidly progressive and life-threatening infection. This study aimed to investigate the clinical characteristics and mortality- associated factors in diabetic patients.

**Methods:**

Detailed clinical information of 165 NF cases was retrospectively collected and analyzed in National Taiwan University Hospital between January 1997 and February 2013. We documented and compared the clinical features according to the presence of underlying diabetes mellitus, and we identified risk factors associated with mortality.

**Results:**

There were 84 patients (51 %) with diabetes. The overall case fatality rate was 29.7 %, and we found no significant difference between the patients with or without diabetes. Compared with the nondiabetic patients, diabetic patients were older and exhibited higher serum levels of glucose and potassium on admission. Polymicrobial infection and monomicrobial NF caused by *Klebsiella pneumoniae* were also more frequently associated with diabetic patients. Moreover, diabetic NF patients exhibit a significantly higher chance of limb loss during hospitalization. In the combined diabetic and nondiabetic cohort, a high serum level of potassium (odds ratio, 2.2; 95 % confidence interval, 1.2 to 4.02; *P* = 0.011) on admission was independently associated with mortality, whereas positive blood culture on admission was associated with mortality in the diabetic cohort (odds ratio, 7.36; 95 % confidence interval, 1.66 to 32.54; *P* = 0.009).

**Conclusions:**

Diabetic patients are more susceptible to NF caused by polymicrobial infection or *K. pneumoniae*, and they are more likely to receive limb amputation for infection control. Bacteraemia on admission is a significant risk factor for mortality in diabetic NF patients.

## Background

Necrotizing fasciitis (NF) has been defined as a severe soft-tissue infection that causes extensive necrosis of subcutaneous tissue and fascia, relatively sparing the muscle and skin tissue [1]. As the disease progresses, thrombosis of the affected cutaneous perforators subsequently devascularizes the overlying skin, causing skin necrosis and haemorrhagic bullae to form. Bacteraemia and sepsis invariably develop when the infection is well established [2]. Despite aggressive treatment, the reported case fatality rate for NF remains high at a cumulative rate up to 34 % [3]. In the literature, most NF patients have preexisting medical conditions, including gout, peripheral arterial occlusive disease, myelodysplastic syndrome, liver cirrhosis, and other immunosuppressive conditions [4–7]. Diabetes mellitus (DM) has been reported to be a common underlying disease in NF patients, accounting for 44.5–72.3 % in various series [1, 6, 8, 9]. Diabetic patients exhibit impaired cutaneous wound healing and increased susceptibility to infection, which may affect the course of soft-tissue infections [10]. It is thus reasonable to speculate that this chronic, debilitating disease contributes to a more serious nature of NF.

Based on bacterial culture results, NF is classified into the following categories: type I, which consists of synergistic polymicrobial infection; type II, representing infections caused by group A *Streptococcus* alone or combined with S*taphylococcus*; and type III, which comprises infections caused by *Vibrio* species [11]. However, recent studies have revealed emerging monomicrobial pathogens of NF, such as methicillin-resistant *Staphylococcus aureus* (MRSA), indicating that the bacteriology of the causative agent of NF is constantly changing [12–14]. Moreover, certain causative agents of NF, including *Klebsiella pneumonia*, are more likely to involve underlying immunocompromising conditions [8]. Therefore, exploration of the bacteriology of NF patients with specific underlying conditions is of great value in guiding the empirical antimicrobial therapy.

Although previous studies have attempted to investigate the clinical characteristics of NF in diabetic patients [15–17], little is known about the microbiology profile and the mortality-associated factors of these patients. Hence, this study was designed to compare the clinical features, including patient age, comorbidities, extent of infection, microbiology, laboratory markers, and time to surgery, in diabetic and nondiabetic NF patients, and we also explored the possible risk factors associated with mortality.

## Methods

### Study population

Using the International Classification of Diseases (ICD)-9 code of NF (728.86), we identified all consecutive NF cases confirmed by surgery between January 1997 and February 2013 in National Taiwan University Hospital, which is a university-affiliated medical centre featuring a 2200-bed capacity. The hospital provides both primary and tertiary referral care in Northern Taiwan. The study procedures were approved by a Research Ethics Committee of National Taiwan University Hospital (201310011RIND). The ethics committee waived the need for obtaining consent from the patients, and the patient data from the dataset were anonymized for further analysis.

### Definition of clinical variables

A definitive diagnosis of NF was based on the intraoperative and histopathological findings. Operative findings are characterised by a lack of resistance to blunt dissection of the normally adherent fascia and the presence of necrotic fascia with purulent malodorous discharge. Diagnosis of diabetes was made based on clinical history and past laboratory tests according to the 2010 American Diabetes Association criteria [18]. Data on the causal microorganisms were collected by reviewing the microbiological reports of wound and/or blood culture samples that were obtained before or at the time of operation. Other clinical variables were defined according to the current literature (Table 1) [8, 19].

**Table 1:** Definitions of clinical parameters

Variable	Definition
Diabetes mellitus	The diagnosis is based on one of four abnormalities outlined by American Diabetes Association, 2010: glycated hemoglobin (A1C) ≥6.5 %, fasting plasma glucose (FPG) ≥126 mg/dl, random plasma glucose ≥200 mg/dl with symptoms, or two-hour plasma glucose ≥200 mg/dl during an oral glucose tolerance test (OGTT)
Functional status	Patient requiring no, partial or totally dependent assistance from another person for activities of daily living before surgery
Hypertension	Patient with elevated blood pressure requiring medication
Chronic renal failure	Patient with a rising creatinine >3 mg/dl or a decreased glomerular filtration rate of less than 60 ml/min/1.73 m^2^ for 3 or more months
Liver cirrhosis	Diagnosis based on clinical, laboratory and echographic findings
SIRS	Includes the presence of two or more of the following: temperature >38 °C or <36 °C, heart rate >90 bpm, respiratory rate >20 breaths/min or PaCO_2_ < 32 mm/Hg, WBC >12,000/mm^3^ or <4000/mm^3^, or >10 % immature (band) forms, anion gap acidosis
Septic shock	Includes sepsis and documented organ and/or circulatory dysfunction
ASA score	1 = normal healthy patient
	2 = patient with mild systemic disease
	3 = patient with severe systemic disease
	4 = patient with severe systemic disease that is a constant threat to life
	5 = moribund patient who is not expected to survive without the operation
Limb loss	Amputation of the lower or upper limb above the ankle or the wrist, respectively.

*SIRS* systemic inflammatory response syndrome, *ASA* American Society of Anesthesiologists

### Treatment protocol

The treatment protocol included emergent radical surgery, broad-spectrum antibiotic therapy, aggressive resuscitation, and soft tissue reconstruction. Emergent surgery with endotracheal tube insertion and general anesthesia was performed in all patients. Empirical antibiotic therapy with oxacillin and a third-generation cephalosporin was usually prescribed upon suspicion of NF. The antibiotic regimen was adjusted when the culture results were available. Moreover, aggressive resuscitation, including intravenous fluid and inotropic agents, were given to all patients to maintain mean arterial pressure above 65 mmHg [13].

### Collection of clinical data

We used a computerised data collection form to systematically collect the following information from the medical records of NF patients:

1. clinical manifestations, including the infection site(s), local findings, severity of sepsis syndrome, and laboratory data on admission;
2. underlying medical conditions, including DM, malignancy, liver cirrhosis, use of immunosuppressants, and chronic renal failure;
3. laboratory results in the emergency room or within 24 h after admission; haemoglobin, white blood cell count, platelet count, sodium, and potassium were expressed as continuous variables, and C-reactive protein and glucose were dichotomised using a cut-off point that was based on clinical experience and previous reports [19];
4. treatments and outcomes, including time to surgery, surgical interventions, limb loss, hospital stay, and in-hospital mortality.

### Statistical analysis

The data were summarized using descriptive statistics. For categorical variables, descriptive analysis were based on percentages and frequencies, and the continuous variables are expressed as the mean and standard deviation (SD). For between-group comparison, the Student’s *t* test was used for continuous data and the chi-square test or Fisher’s exact test was used for categorical data. The potential risk factors for mortality were examined in all of the NF patients, diabetic NF patients, and nondiabetic NF patients. Logistic regression was performed to produce odds ratios (ORs) with 95 % confidence intervals (CIs). Multivariate logistic regression was used in assessing risk factors by adding forward substitution factors identified as significant in the univariate analysis (*P* <0.05). All reported *P* values were two-tailed and *P* <0.05 indicated statistical significance in each analysis. The statistical analyses were performed using SAS 9.2 (SAS Institute Inc., Cary, NC).

## Results

### General clinical characteristics

We identified 165 NF patients that met the inclusion criteria. The mean age was 58.8 years, and 110 patients were men (67 %) and 55 patients were women (33 %). Underlying chronic diseases, including DM (*n* = 84, 51 %), hypertension (*n* = 59, 36 %), heart disease (*n* = 39, 24 %), chronic kidney disease (*n* = 29, 18 %), and liver cirrhosis (*n* = 22, 13 %) were common. The infection most commonly involved the lower limbs (*n* = 111, 67 %), followed by the trunk (*n* = 21, 13 %), and the upper limbs (*n* = 17, 10 %). Seventy-eight patients (47 %) reported a preceding trauma or injury history at the infection site. Antibiotic susceptibility data of the causative agents were available in 81 patients, and 19 of them (23.5 %) were found to receive inappropriate empirical antibiotics. During hospitalisation, the NF patients underwent an average of 2.7 ± 1.8 surgical debridements. Major limb amputation was performed in 35 patients (21 %), and 49 patients died during hospitalization, yielding a case fatality rate of 29.7 %.

### Comparison of diabetic and nondiabetic NF patients

Among the 165 NF cases, 121 (73.3 %) were monomicrobial, 30 (18.2 %) were polymicrobial, and 14 (8.5 %) were culture negative (Table 2). The most common pathogens of monomicrobial NF were *K. pneumoniae* (*n* = 17), group A *Streptococcus* (*n* = 16), and MRSA (*n* = 12). Causative agents of NF in the diabetic patients were more likely to be polymicrobial (*P* < 0.01), whereas significantly more monomicrobial NF infections caused by *K. pneumoniae* developed in diabetic patients (DM, 13 cases vs. non-DM, 4 cases*, P* < 0.05).

**Table 2:** Causative bacteria cultured from 165 necrotizing fasciitis cases

NF type, Causative pathogen(s)	All patients, n (%)	DM, n (%)	Non-DM, n (%)	*P* value (DM vs non-DM)
Monomicrobial				
Group A *Streptococcus*	12 (7.3)	3 (3.6)	9 (11.1)	0.076
Group B *Streptococcus*	4 (2.4)	2 (2.4)	2 (2.5)	1.0
*Staphyloccocus aureus*				
MRSA	14 (8.5)	6 (7.1)	8 (9.9)	0.529
MSSA	12 (7.3)	5 (6.0)	7 (8.6)	0.561
*Aeromonas hydrophilia*	7 (4.2)	2 (2.4)	5 (6.2)	0.271
*Vibrio vulnificus*	9 (5.5)	4 (4.8)	5 (6.2)	0.737
*Escherichia coli*	10 (6.1)	4 (4.8)	6 (7.4)	0.530
*Klebsiella pneumoniae*	17 (10.3)	13 (15.5)	4 (4.9)	0.038
*Pseudomonas aeruginosa*	4 (2.4)	0 (0)	4 (4.9)	0.056
*Serratia marcescens*	4 (2.4)	2 (2.4)	2 (2.5)	1.0
Others	28 (17.0)	14 (16.7)	14 (17.3)	0.916
Polymicrobial				
Mixed infection	30 (18.2)	22 (26.2)	8 (9.9)	0.007
Culture negative				
No bacterial growth	14 (8.5)	7 (8.3)	7 (8.6)	0.943
Total	165	84	81	

*DM* diabetes mellitus, *MRSA* methicillin-resistant *Staphylococcus aureus*, *MSSA* methicillin-sensitive *Staphylococcus aureus*

We classified the NF patients into DM and non-DM cohorts and compared their clinical characteristics (Table 3). Relative to the nondiabetic patients, the diabetic patients were significantly older (*P* < 0.05) and presented with significantly higher serum levels of glucose and potassium upon admission (*P* < 0.01). Although the case fatality rate of NF was comparable between the DM and non-DM cohorts (DM, 28.6 % vs. non-DM, 30.9 %, P = 0.747), the limb amputation rate was significantly higher among the diabetic NF patients (DM, 28.6 % vs. non-DM, 13.6 %, *P* <0.05).

**Table 3:** Clinical characteristics of the non-diabetic and diabetic cohorts among necrotizing fasciitis patients

Variable	DM (*n* = 84)	Non-DM (*n* = 81)	*P* value
Age	61.5 ± 10.6	56 ± 22.2	0.045
Male gender	58(69.1)	52(64.2)	0.509
American Society of Anaesthesiologists (ASA) score			
1	4(4.8)	14(17.3)	0.079
2	23(27.4)	20(24.7)	
3	37(44.0)	32(39.5)	
4	20(23.8)	15(18.5)	
Pre-existing medical conditions			
Hypertension	35(42.2)	24(29.6)	0.094
Chronic renal failure	19(22.6)	10(12.4)	0.083
Liver cirrhosis	12(14.3)	10(12.4)	0.714
Malignancy	6(7.1)	8(9.9)	0.529
Immunosuppresant medication	7(8.3)	8(9.9)	0.730
Admission lab data			
Hemoglobin (mg/dl)	10.4 ± 2.5	10.8 ± 2.5	0.321
Leukocyte count (10^3^/mm^3^)	11.1 ± 9.9	10.5 ± 11.8	0.767
Platelet count (10^3^/mm^3^)	188.8 ± 129.5	160.7 ± 109.3	0.167
C-reactive protein > 10 mg/dl^a^	43(75.4)	44(83.0)	0.329
Glucose > 110 mg/dl^b^	41(85.4)	17(48.6)	<0.001
Potassium (mEq/l)	4.4 ± 1.1	4.0 ± 0.8	0.010
Positive blood culture^c^	28(41.8)	27(49.1)	0.420
Location of infection			
body	10(11.9)	11(13.6)	0.651
head and neck	3(3.6)	1(1.2)	
upper limb	9(10.7)	8(9.9)	
lower limb	54(64.3)	57(70.4)	
multiple sites	8(9.5)	4(4.9)	
Time to surgery <12 h	43(51.2)	44(54.3)	0.687
ICU stay (day)	9.3 ± 10.8	9.8 ± 12.1	0.793
Hospital stay (day)	39.1 ± 28.6	41.1 ± 38.4	0.712
Limb loss	24(28.6)	11(13.6)	0.019
Mortality	24(28.6)	25(30.9)	0.747

### Risk factors for mortality

We also compared the clinical characteristics of the survivors and non-survivors among NF patients (Table 4). Then mortality-associated risk factors were determined in diabetic and nondiabetic NF patients as a combined cohort, and then separately. No significant differences in the parameters of age, gender, site of involvement, types of NF infection, appropriate empirical antibiotic use, and time to first surgical intervention were observed between survivors and non-survivors in either the combined or respective cohort. For all of the NF patients, clinical signs of dyspnea and hypotension upon admission were associated with mortality according to a univariate analysis. Moreover, initial laboratory data revealing a low platelet count, a high blood level of potassium, and a positive blood culture were also mortality-associated factors. According to a multivariate analysis, only a high blood level of potassium upon admission was independently associated with mortality (Table 5).

**Table 4:** Clinical characteristics of the survivors and non-survivors among necrotizing fasciitis patients

Variable	Survivors (*n* = 116)	Non-survivors (*n* = 49)	*P* value
Age	57.8 ± 17.9	61 ± 16.4	0.297
Male gender	75(64.7)	35(71.4)	0.399
American Society of Anaesthesiologists (ASA) score			
1	13(11.2)	5(10.2)	0.268
2	33(28. 5)	10(20.4)	
3	50(43.1)	19(38. 8)	
4	20(17.2)	15(30.6)	
Pre-existing medical conditions			
Diabetes mellitus	60(51.7)	24(49.0)	0.747
Hypertension	39(33.6)	20(41. 7)	0.329
Chronic renal failure	17(14.7)	12(24.5)	0.129
Liver cirrhosis	13(11.2)	9(18.4)	0.216
Malignancy	10(8.6)	4(8.2)	1
Immunosuppresant medication	9(7.8)	6(12.2)	0.382
Admission lab data			
Hemoglobin (mg/dl)	10.8 ± 2.4	10.1 ± 2.6	0.135
Leukocyte count (10^3^/mm^3^)	11.3 ± 11.3	9.7 ± 9.9	0.3777
Platelet count (10^3^/mm^3^)	193.7 ± 128.8	135.2 ± 89.0	0.0021
C-reactive protein > 10 mg/dl^a^	58(79. 5)	29(78.4)	0896
Glucose > 110 mg/dl^b^	39(65)	19(82.61)	0.356
Potassium (mEq/l)	4.0 ± 0.9	4.5 ± 1.2	0.034
Positive blood culture^c^	33(38.82)	22(59.46)	0.035
Location of infection			
body	9(7.8)	12(24.5)	0.025
head and neck	3(2.6)	1(2.0)	
upper limb	10(8.6)	7(14.3)	
lower limb	84(72.4)	27(55.1)	
multiple sites	10(8.6)	2(4.1)	
Time to surgery <12 h	62(54.39)	25(54.35)	0.531
ICU stay (day)	9.5 ± 12.1	9.5 ± 10.4	0.977
Hospital stay (day)	51.7 ± 33.4	13.6 ± 14.4	<.0001
Limb loss	28(24.1)	7(14.3)	0.157

In Tables 3 and 4, data presented as mean ± standard deviation for continuous data or number (%) for categorical data

^a^data derived from 103 of the 165 cases

^b^data derived from 72 of the 165 cases

^c^data derived from 114 of the 165 cases

**Table 5:** Univariate & multivariate analysis of risk factors for mortality in all necrotizing fasciitis patients

Variable	Univariate analysis	Multivariate analysis		
	OR(95 % CI)	*P* value	OR(95 % CI)	*P* value
Age	1.01(0.99,1.03)	0.3		
Male gender	1.37(0.66,2.83)	0.4		
Functional status				
Partially dependent	1	-		
Totally dependent	0.79(0.25,2.52)	0.69		
Totally independent	0.79(0.38,1.61)	0.51		
Underlying medical conditions				
DM	0.90(0.46,1.75)	0.75		
Chronic renal failure	1.89(0.82,4.33)	0.13		
Immunosuppresant medication	1.66(0.56,4.94)	0.36		
Clinical presentation at admission				
Consciousness disturbance	1.77(0.88,3.56)	0.11		
Temperature > 38 °C	1.58(0.77,3.22)	0.21		
Dyspnea	2.68(1.32,5.45)	0.007	2.29(0.81,6.43)	0.117
Systolic BP < 90 mmHg	2.03(1.02,4.06)	0.045	1.14(0.4,3.2)	0.810
SIRS	2.08(0.73,5.88)	0.17		
Pus at infection site	0.73(0.31,1.68)	0.45		
Admission lab data				
Hemoglobulin (mg/dl)	0.90(0.78,1.04)	0.14		
Leukocyte (10^3^/mm^3^)	0.99(0.95,1.02)	0.38		
Platelet (10^3^/mm^3^)	0.995(0.99,0.999)	0.008	1(0.99,1)	0.056
Potassium (mEq/l)	1.57(1.06,2.31)	0.023	2.2(1.2,4.02)	0.011
Positive blood culture	2.31(1.05,5.08)	0.037	1.95(0.71,5.38)	0.197
Time to surgery <12 h	0.83(0.41,1.68)	0.598		

(*n* = 165)

*OR* odds ratio, *CI* confidence interval, *SIRS* systemic inflammatory response syndrome

In the cohort of 84 diabetic NF patients, clinical signs of dyspnea and hypotension upon admission were associated with mortality according to a univariate analysis. Moreover, initial laboratory data revealing a higher level of potassium in the blood and a positive blood culture were also mortality-associated factors. According to a multivariate analysis, only a positive blood culture upon admission was independently associated with mortality in diabetic NF patients (Table 6).

**Table 6:** Univariate & multivariate analysis of risk factors for mortality in diabetic necrotizing fasciitis patients

Variable	Univariate analysis	Multivariate analysis		
	OR(95 % CI)	*P* value	OR(95 % CI)	*P* value
Age	0.98(0.93,1.02)	0.34		
Male gender	2.05(0.67,6.26)	0.21		
Functional status				
Partially dependent	1	-		
Totally dependent	2.72(0.5,14.72)	0.25		
Totally independent	1.81(0.65,5.07)	0.26		
Underlying medical conditions				
Chronic renal failure	1.65(0.56,4.87)	0.37		
Immunosuppresant medication	3.80(0.78,18.47)	0.10		
Clinical presentation at admission				
Consciousness disturbance	1.69(0.64,4.45)	0.29		
Temperature > 38 °C	1.87(0.68,5.18)	0.23		
Dyspnea	3.50(1.24,9.89)	0.018	4.19(0.98,17.91)	0.053
Systolic BP < 90 mmHg	2.89(1.05,7.96)	0.040	1.81(0.45,7.21)	0.403
SIRS	0.78(0.21,2.88)	0.70		
Pus at infection site	1.75(0.59,5.21)	0.31		
Admission lab data				
Hemoglobulin (mg/dl)	0.86(0.69,1.06)	0.16		
Leukocyte (10^3^/mm^3^)	1.01(0.96,1.06)	0.81		
Platelet (10^3^/mm^3^)	0.996(0.99,1.001)	0.13		
Potassium (mEq/l)	1.84(1.08,3.15)	0.025	1.65(0.84,3.24)	0.147
Positive blood culture	4.57(1.52,13.78)	0.007	7.36(1.66,32.54)	0.009

(*n* = 84)

According to univariate analysis of 81 nondiabetic NF patients, an independent functional status and the presence of pus at infection sites were inversely associated with mortality, whereas clinical presentation with *systemic inflammatory response syndrome* (SIRS) and a low platelet count upon admission were associated with mortality. With further multivariate analysis, an independent functional status and the presence of pus at infection sites were independent factors inversely associated with mortality, and the presence of SIRS upon admission was associated with mortality (Table 7).

**Table 7:** Univariate & multivariate analysis of risk factors for mortality in nondiabetic necrotizing fasciitis patients

Variable	Univariate analysis	Multivariate analysis		
	OR(95 % CI)	*P* value	OR(95 % CI)	*P* value
Age	1.02(1.00,1.04)	0.1		
Male gender	0.99(0.37,2.64)	0.98		
Functional status				
Partially dependent	1	-		
Totally dependent	0.22(0.04,1.25)	0.09	0.23(0.03,2.09)	0.193
Totally independent	0.29(0.1,0.85)	0.025	0.16(0.04,0.74)	0.019
Underlying medical conditions				
Chronic renal failure	2.55(0.67,9.77)	0.17		
Immunosuppresant medication	0.73(0.14,3.87)	0.71		
Clinical presentation at admission				
Consciousness disturbance	1.97(0.70,5.56)	0.2		
Temperature > 38 °C	1.40(0.50,3.90)	0.52		
Dyspnea	2.15(0.80,5.76)	0.13		
Systolic BP < 90 mmHg	1.45(0.56,3.78)	0.45		
SIRS	8.21(1.01,66.85)	0.049	12.21(1.09,137.21)	0.043
Pus at infection site	0.22(0.05,1.03)	0.05	0.09(0.01,0.86)	0.037
Admission lab data				
Hemoglobulin (mg/dl)	0.93(0.77,1.13)	0.45		
Leukocyte (10^3^/mm^3^)	0.97(0.93,1.02)	0.18		
Platelet (10^3^/mm^3^)	0.99(0.987,0.999)	0.021	0.997(0.99,1.004)	0.382
Potassium (mEq/l)	1.39(0.71,2.70)	0.34		
Positive blood culture	1.05(0.33,3.37)	0.93		

(*n* = 81)

## Discussion

NF is likely to develop in immunocompromised patients with underlying medical conditions, such as alcohol abuse, malignancy, chronic cardiac and renal disease, intravenous drug usage, immunosuppressive therapy, and malnutrition [9]. Diabetes is a disorder that adversely affects the immune system, thus representing a common underlying disease in NF patients. In our series, the diabetic NF patients were significantly older than the nondiabetic patients at the onset of NF. Moreover, diabetic patients were significantly more likely than nondiabetic patients to exhibit a high blood level of glucose upon admission. Insulin therapy for glycaemic control at a target blood glucose level of 140 to 200 mg/dl has been suggested in critically ill patients [20]. Therefore, the high incidence of hyperglycaemia observed upon admission among diabetic NF patients warrants initiating monitor of blood glucose level and glycaemic control immediately after hospitalisation.

Traditionally, type 1 (polymicrobial) NF was the most common type of infection, emphasizing the synergetic effects of multiple pathogens [2, 3]. Analysis of the causative agents of NF revealed that significantly more diabetic patients exhibited polymicrobial infections than did nondiabetic patients, possibly reflecting the susceptibility of diabetic patients to multiple pathogens [21]. However, single organism infection accounted for the majority of NF (74 %) in this study, which is in line with the recent studies demonstrating a trend of increasing monomicrobial NF [8, 14, 22]. Among the monomicrobial NF cases, we observed significantly more *K. pneumoniae*-related infections in the DM group than in the non-DM group. We previously described *K. pneumoniae* as a common pathogen of monomicrobial NF in Taiwan, particularly in patients with underlying host immunocompromising conditions [8]. The present study further emphasized the importance of recognizing monomicrobial NF caused by *K. pneumoniae* as a common entity of NF in diabetic patients. Therefore, initial empirical antimicrobial agents for NF should be considered depending on the presence of underlying diabetes.

In our series, a non-significant difference in case fatality rate between diabetic and nondiabetic patients was observed. This is in line with the finding of previous studies that DM combined with other comorbidities was associated with an increased risk of death, but DM alone was not a risk factor for mortality [4, 17]. Also consistent with many previous studies, NF was most commonly involved in the lower limbs in this study [6, 8, 9]. Moreover, we found that significantly more diabetic patients than nondiabetic patients experienced limb loss during the treatment course. Diabetic patients are well known to be afflicted with foot problems caused by neuropathy and microvascular disease, which render limb preservation more difficult in the presence of a superimposed NF infection. Amputation typically requires less time than radical debridement of the necrotic soft tissue, and requires few, if any, reconstructive procedures [4]. Although major limb amputation deprives the amputee of motional dexterity and should be avoided whenever possible, NF is one of the few exceptions for which this extreme procedure should be considered to save lives [23].

Multiple patient- and treatment-related risk factors for NF-associated mortality have been described [24–27]. However, the identified risk factors among various studies exhibited some discrepancies. For example, some studies linked poor outcomes to delays in surgical management [6, 28], whereas other studies, similar to this one, found delayed surgical management did not significantly impact mortality [19, 27]. The discrepancies among different studies reflect the diversity of the populations included and the variables studied. In this study, we focused on factors that can be easily identified at the initial presentation of NF patients.

In all NF cases in this series, mortality was associated with a high level of serum potassium on admission. Electrolyte imbalance is a common clinical presentation of NF [6, 29], and Ogilvie et al. also showed an increased risk of mortality in NF patients with elevated potassium levels [30]. The elevated serum potassium level on admission may reflect acute renal impairment. Unfortunately, serum creatinine level was not collected in our database so the association between hyperkalemia and renal impairment could not be confirmed. Nonetheless, our finding still highlights the importance of the supportive care with fluid and electrolyte management in NF patients. Further subgroup analysis revealed that a positive blood culture upon admission was associated with mortality in diabetic NF patients. Bacteraemia frequently causes sepsis, which is the typical cause of death in NF patients. As virulent organisms and toxins are released into the bloodstream from severely infected soft tissue, a systemic toxic reaction is initiated, resulting in hypotension, disseminated intravascular coagulation, and eventually multiorgan failure.

The presence of SIRS upon admission was also identified as a mortality- associated factor in nondiabetic NF patients. Moreover, a completely independent functional status and the presence of pus at the infection site upon admission were associated with a more favourable chance of survival in the non-DM group. The functional status reflects the general physical condition of a person, thus an independent functional status can reasonably serves as a predictor of better outcome. However, the association between the presence of pus upon admission and a favourable outcome is intriguing. Similarly, a previous study identified the presence of hemorrhagic bullae on admission as an independent negative predictor of mortality in NF patients [22]. Diagnosis of NF at an early stage is often difficult and depends on a high index of suspicion because early cutaneous findings, including edema, erythema, and local anaesthesia, are typically nonspecific [6, 29]. Therefore, we speculated that obvious cutaneous manifestations, such as pus or bullae formation, at the infection sites may prompt surgical consultation, necessitating early diagnosis and immediate surgical intervention, which are crucial for the survival of NF patients.

The limitations of this study are attributable to the retrospective study design and possible inaccuracy of the information retrieved from the medical records or the possible misinterpretation of this information. However, we attempted to address these problems by setting clear definition for variables prior to data collection. We also excluded variables without sufficient data from further statistical analysis, such as arterial blood gas and serum albumin level. Moreover, not all of the factors related to NF prognosis, such as nutrition, surgical dexterity, and postoperative wound care, could be considered. To enhance our understanding of NF, a prospective registry for all NF patients confirmed according to surgery will be a valuable tool [9]. It may include standard recording of underlying diseases, presenting symptoms and signs, clinical photography, thorough laboratory data of blood samples, imaging and bacteriological studies, and treatment course.

## Conclusions

Diabetes is a common predisposing medical condition of NF, and it is associated with a significantly higher chance of limb loss. Relative to nondiabetic patients, diabetic NF patients were older and presented with a higher serum level of glucose and potassium. Moreover, diabetic NF patients were more susceptible to polymicrobial and monomicrobial *K. pneumoniae* infections, which should be considered when choosing empirical antibiotics for these patients. Bacteraemia on admission is significant risk factor for mortality in NF patients with diabetes. We also identified several independent risk factors for mortality in nondiabetic NF patients. These information can be applied in predicting outcomes and used to guide the treatment of NF patients according to the presence of underlying diabetes. This study thus provides an impetus for further clinical research to assess the impact of modification of these identified factors on the subsequent clinical outcomes of both diabetic and nondiabetic NF patients.

## Abbreviations

NF: Necrotizing fasciitis
DM: Diabetes mellitus
MRSA: Methicillin-resistant *Staphylococcus aureus*
SD: Standard deviation
OR: Odds ratio
CI: Confidence interval
SIRS: Systemic inflammatory response syndrome
